# Tissue-specific micropattern array chips fabricated via decellularized ECM for 3D cell culture

**DOI:** 10.1016/j.mex.2023.102463

**Published:** 2023-10-31

**Authors:** Xinglong Zhu, Yi Li, Hulin Long, Zuoyu Liang, Yuting He, Yanyan Zhou, Shun Li, Ji Bao

**Affiliations:** a Department of Pathology, Institute of Clinical Pathology, Key Laboratory of Transplant Engineering and Immunology, West China Hospital, Sichuan University, Chengdu, Sichuan 610041, China; b Institute of Respiratory Health, West China Hospital, Sichuan University, Chengdu, Sichuan 610041, China; c Precision Medicine Key Laboratory, West China Hospital, Sichuan University, Chengdu, Sichuan 610041, China; d Department of Rehabilitation Medicine, Hospital of Chengdu University of Traditional Chinese Medicine, Chengdu, Sichuan 610041, China; e Department of Biophysics, School of Life Science and Technology, University of Electronic Science and Technology of China, Chengdu, Sichuan 610054, China

**Keywords:** Micropatterned arrays, Decellularized extracellular matrix, Cell spheroids, Organoids, The tissue specific micropattern array chips fabricated via dECM for 3D cell culture

## Abstract

Multicellular three-dimensional (3D) in vitro models, such as cell spheroids and organoids, can significantly improve the viability, histomorphology, genotype stability, function and drug metabolism of cells [1], [2], [3]. In general, several culture methods of 3D models, including the hanging drop, microwell-mesh and hydrogel encapsulating methods, have difficulty building a standard mode and controlling the size and arrangement of cell spheroids or organoids, which could severely affect the authenticity and repeatability of experimental results [4]. Another key factor in 3D in vitro models is the extracellular matrix (ECM), which can determine cell viability, proliferation, differentiation, function, migration and organization [5]. In this study, micropattern array chips combined with decellularized ECM (dECM) not only provide tissue-specific ECM but also control the size and arrangement of 3D models.

• Methods have been established to demonstrate the use of dECM as a bioink to generate dECM-coated micropattern array chips by microcontact printing.
• The micropattern can limit cell growth and migration, and cells spontaneously assemble into cell spheroids with uniform size and orderly arrangement.

## Graphical abstract

**Figure:**
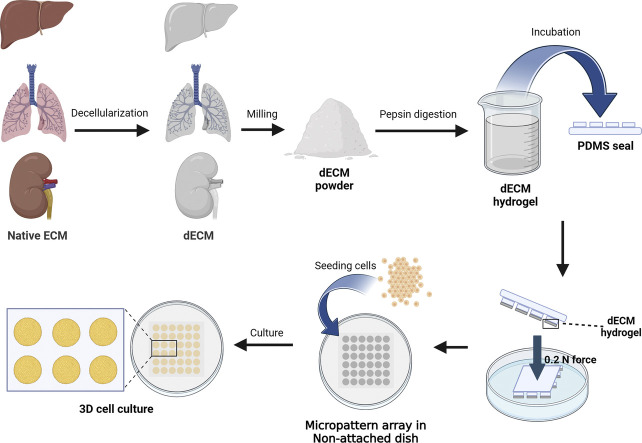

Specifications table

**Table:** 

Subject area:	Engineering
More specific subject area:	Biomedical engineering
Name of your method:	The tissue specific micropattern array chips fabricated via dECM for 3D cell culture
Name and reference of original method:	Zhu, X., Li, Y., Yang, Y., He, Y., Gao, M., Peng, W., Wu, Q., Zhang, G., Zhou, Y., Chen, F., Bao, J., & Li, W. (2022). Ordered micropattern arrays fabricated by lung-derived dECM hydrogels for chemotherapeutic drug screening. *Materials today. Bio, 15*, 100274. https://doi.org/10.1016/j.mtbio.2022.100274
Resource availability:	NA

## Method details

### Preparation of the dECM

• Male Bama miniature pigs weighing 30–40 kg were purchased and kept in biosecure facilities from Sainuo Biomedical (Chengdu, China). Pigs were anesthetized with Zoletil 50 (10 mg/kg body weight, Virbac, France) and maintained with propofol (6 mg/kg/h, Qingyuan Jiabo, China). The liver, lung and kidney were excised, and the hepatic portal vein of the liver, the pulmonary artery of the lungs and the renal aorta of the kidney were cannulated, respectively. The blood was flushed out with heparin-phosphate buffer saline (PBS) for 10 min and then frozen at -20 °C for further use. Before decelluarization, the liver, lung and kidney were thawed for disruption of the cell structure.
  ➢ Liver: The thawed liver was perfused via the hepatic portal vein with double-distilled water (ddH_2_O) at 200 ml/min for 1 h. For decellularization, 1 % (v/v) Triton X-100 (Sigma–Aldrich, #X100), 1 % (w/v) sodium dodecyl sulfate (SDS, Sigma–Aldrich, #L3771) and 1 % (v/v) Triton X-100 were perfused at 200 ml/min for 3 h, 6 h, and 3 h, respectively. Finally, the ddH_2_O was perfused at 200 ml/min for 3 h for removing these detergents [6].
  ➢ Lung: The trachea of thawed lung was intermittently clamped and then perfused via the pulmonary artery with ddH_2_O at 100 ml/min for 1 h. For decellularization, 1 % (v/v) Triton X-100, 1 % (v/v) sodium lauryl ether sulfate (SLES, Biofroxx) and 1 % (v/v) Triton X-100 were perfused at 100 ml/min for 3 h, 6 h, and 3 h, respectively. Finally, the ddH_2_O was perfused at 100 ml/min for 3 h for removing these detergents [7].
  ➢ Kidney: The thawed kidney was perfused via the renal aorta with ddH_2_O at 15 ml/min for 1 h. For decellularization, 1 % (v/v) Triton X-100, 1 % (v/v) SLES and 1 % (v/v) Triton X-100 were perfused at 15 ml/min for 3 h, 6 h, and 3 h, respectively. Finally, the ddH_2_O was perfused at 15 ml/min for 3 h for removing these detergents [8].
• The decellularized liver, lung and kidney were cut into cubes (1 × 1 × 1 cm) for lyophilization.
• Lyophilized cubes were powdered via the Wiley Mill (Retsch, MM400, Germany) and then stored at −80 °C.
• The dECM powders were digested with 10 % (w/w) pepsin (Sigma–Aldrich) in 0.01 M HCl through stirring at room temperature for 48 h. The dECM hydrogel was then neutralized to a pH of 7.2-7.4 by adding 0.1 M NaOH. Its final concentration was then adjusted to 10 mg/ml using 1 × PBS. The dECM hydrogel was stored at −4 °C as the bioink for further fabrication of tissue-specific micropattern array chips [7].

### Micropattern array printing

**Fig. 1:**
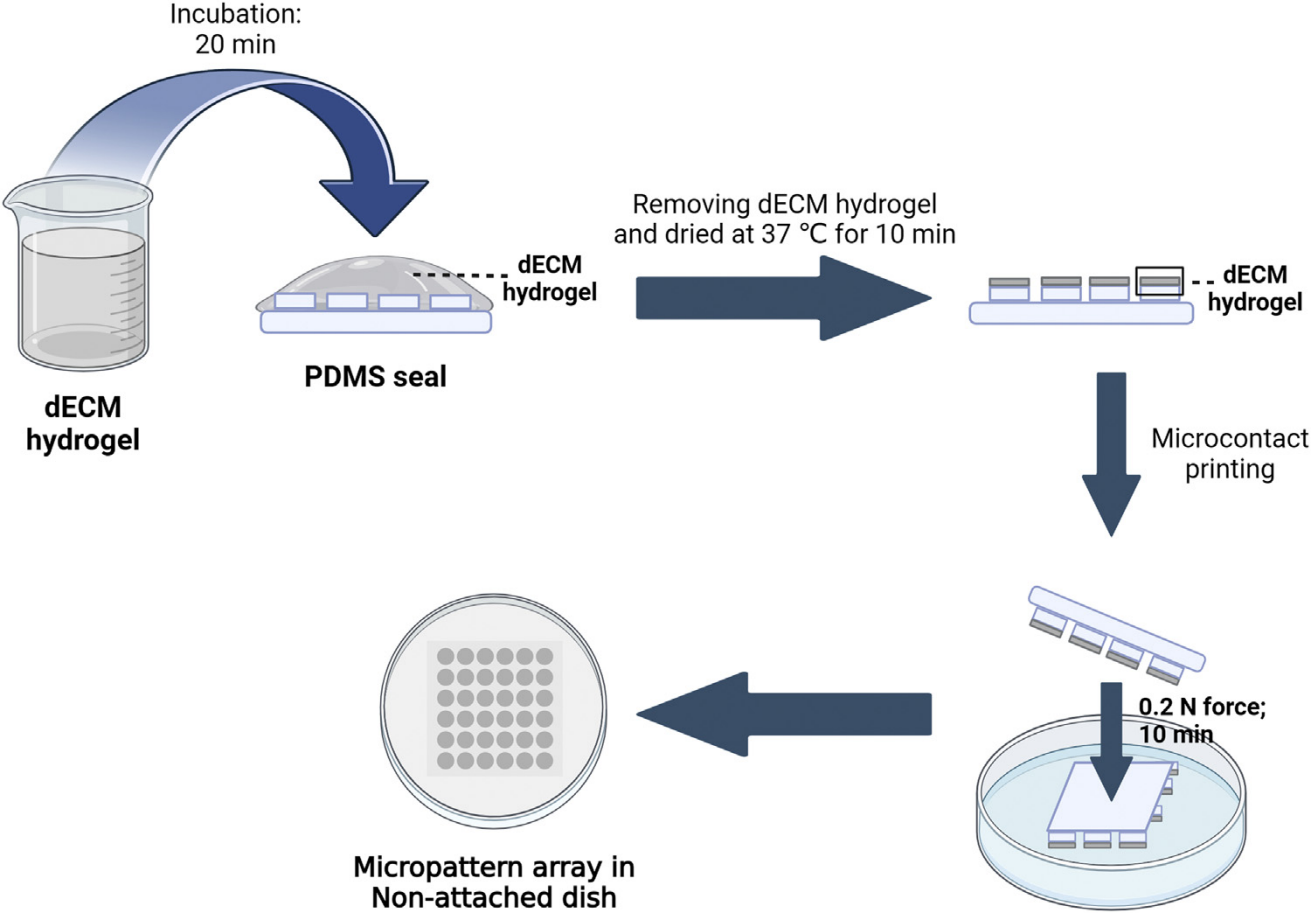
Stepwise demonstration of dECM as a bioink to fabricate tissue-specific micropattern array chips.

• The customed polydimethylsiloxane (PDMS) seals were obtained through laser etching of the characteristic pattern on a silicon wafer. The surface of PDMS seals contained a number of cylinders (diameter: 100 µm; height: 20 µm; spacing between cylinders: 50 µm).
• The surfaces of 1 × 1 or 2 × 2 cm^2^ PDMS seals were coated with 0.2 or 1 ml of 0.1 mg/ml dECM hydrogel with 20 µg/ml fluorescein isothiocyanate isomer (FITC, Sigma) for 20 min, respectively.
• The dECM hydrogel was removed from the surface of the PDMS seals, and then the seals were dried at 37 °C for 10 min.
• The coated seal was placed on a 35-mm diameter nontreated cell culture dish (BIOFIL, TCD000035) at 0.2 N force for 10 min. The seal was removed (Fig. 1).

### Cell spheroid culture

• Cell culture: Human hepatoblastoma HepG2 cells and human lung cancer A549 cells were cultured in high-glucose Dulbecco's modified Eagle's medium (DMEM, HyClone) containing 10 % fetal bovine serum (Royacel) and 1 % penicillin–streptomycin solution (HyClone) and human renal cancer OS-RC-2 cells were cultured in RPMI-1640 medium (HyClone) supplemented with 10 % fetal bovine serum and 1 % penicillin–streptomycin solution in an incubator at 37 °C under 5 % CO_2_ with saturated humidity.
• The dECM-patterned dishes were coated with 10 mg/ml pluronic F-127 (Sigma) at dark for 1 h to prevent nonspecific cellular adherence.
• The dECM-patterned dishes were then sterilized by ultraviolet irradiation for 1 h.
• A total of 1-5 × 10^5^ cells (depending on the kinds of cells) were seeded on dECM-patterned dishes containing 3 ml of culture medium.
• The culture medium was removed after 4-8 h of incubation (depending on the kinds of cells). These dishes were washed three times with PBS to remove the unattached cells. Then, 2.5 ml of medium was added to the dish for cell spheroid culture and changed every other day.
• The growth of cells was observed on dECM-patterned dishes every day.

## Method validation

### The tissue-specific micropattern array chips

**Fig. 2:**
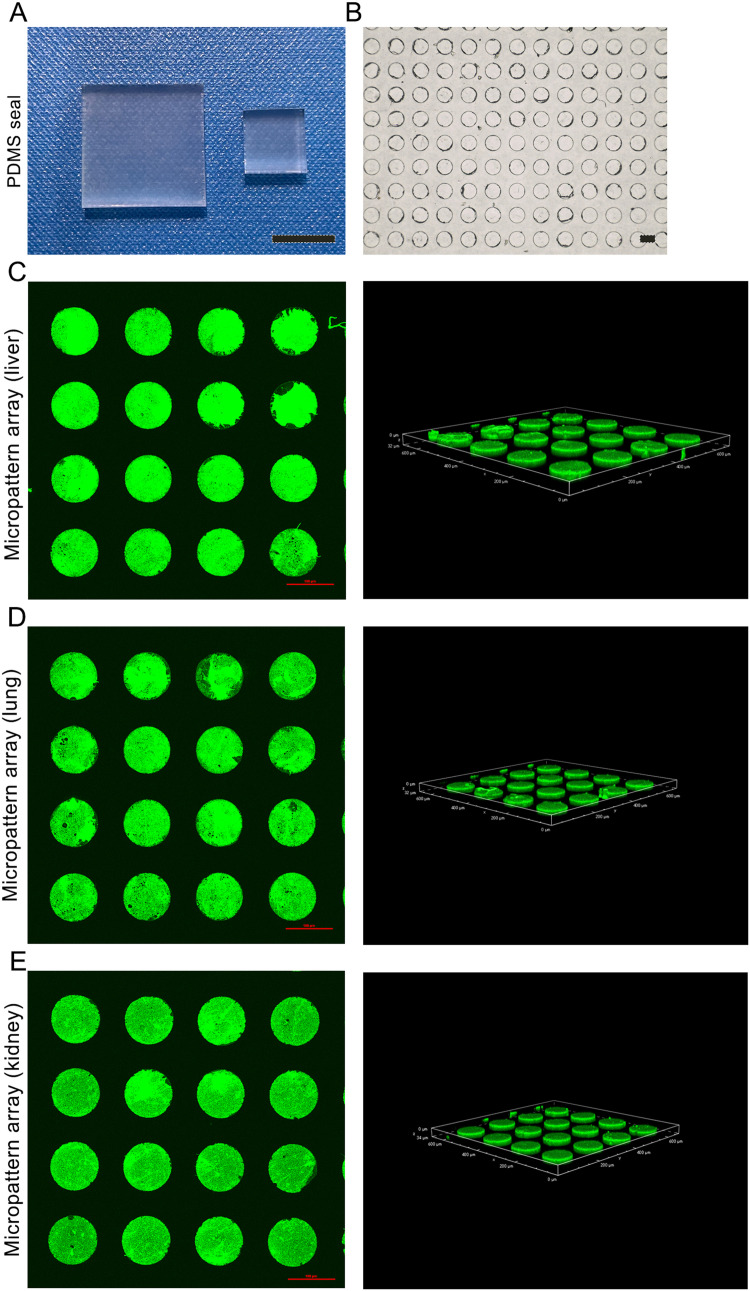
**The fabrication of tissue-specific micropattern array chips.** (A) Macroscopic view of the PDMS seals. (B) The surface of PDMS seals in the bright field. (C) The liver dECM micropattern array in the dish. (D) The lung dECM micropattern array in the dish. (E) The kidney dECM micropattern array in the dish. The scale bar of A = 1 cm; scale bars of B, C, D and E = 100 µm.

As shown in Fig. 2A, 1 × 1 or 2 × 2 cm^2^ PDMS seals were fabricated. In the bright field, the surface of the PDMS seals were uniformly round micropatterns with diameters of 100 µm and 50 µm spacing between micropatterns (Fig. 2B). The dECM hydrogel (liver, lung and kidney) as the bioink was coated on the surface of PDMS seals to generate uniformly dECM-coated round micropattern arrays on the dishes (Figs. 2C-E).

### Cell culture

**Fig. 3:**
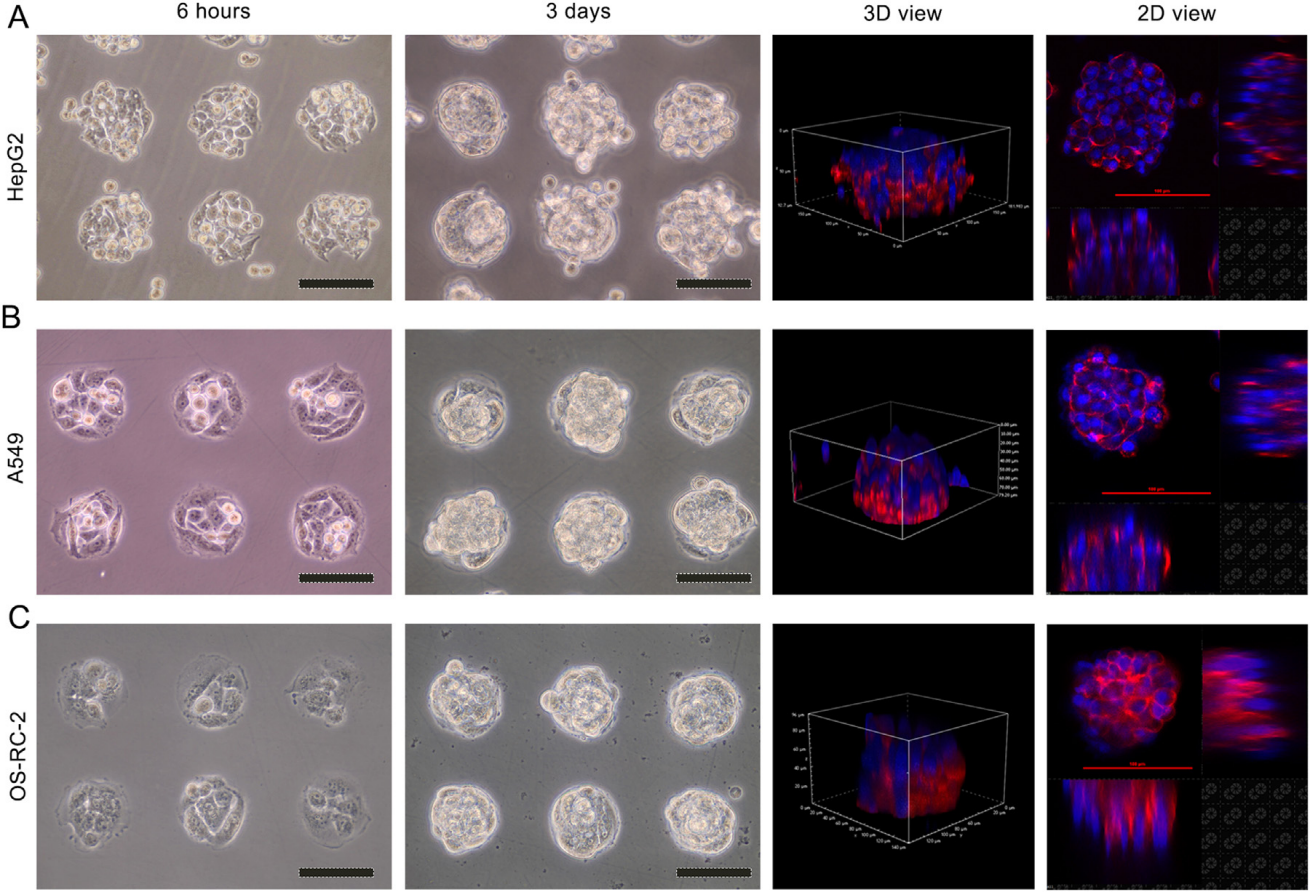
Tissue-specific micropattern array chips for 3D cell culture. (A) HepG2 cells. (B) A549 cells. (C) OS-RC-2 cells. Scale bar = 100 µm.

HepG2 cells (2 × 10^5^), A549 cells (3 × 10^5^) and OS-RC-2 cells (2 × 10^5^) were seeded on sterilized tissue-specific micropattern array chips (liver, lung and kidney). After 6 h of attachment and washing three times, the cells were uniformly attached on the tissue-specific micropattern to form a monolayer of cells (Figs. 3A-C). Consequently, the growth of cells was limited on the micropattern. With prolonged culture time, size-controllable and orderly cell spheroids were gradually fabricated (Figs. 3A-C).

For further observation of the 3D architecture of cell spheroids, the cell spheroids on the dishes were fixed with 4 % formaldehyde for 20 min at room temperature, stained with rhodamine phalloidin solution (PHDR1, 100 nM, Cytoskeleton, CA, USA) for 45 min at room temperature, and counterstained with DAPI. On the 100 µm round micropattern. The HepG2, A549 and OS-RC-2 spheroids showed a 3D spheroid shape (Fig. 3A-C). Therefore, the tissue-specific micropattern array chips could not only support cells to grow cell spheroids but also control the size and arrangement of cell spheroids.

In conclusion, the novel tissue-specific micropattern array chips can efficiently generate cell spheroids. Compared with hanging drop, microwell-mesh and hydrogel encapsulating methods, the chip can effectively control the size and arrangement of cell spheroids to bring convenience to analysis and observation, especially in the drug screening [6,7,9]. Furthermore, Chips can be combined with 3D imaging (confocal imaging, high content imaging and multiphoton microscopy) and commercial automated micro-injection systems to realize automated manipulation and analysis [10], [11], [12]. This cell culture platform could be also used to culture primary cells (infantile hemangioma) to fabricate microtumors [13]. Another advantage of tissue-specific micropattern array chips is that dECM can supply the tissue-specific matrix and microenvironment. As the matrix, bioink and scaffolding material, the dECM was gradually used to culture and regulate primary cells, stem cells and cell lines, construct bioengineering tissues or organs combining 3D printing technology and repair damaged tissues or organs [14]. Chemical synthetic materials (PEG-based materials), natural materials (sodium alginate) and single ECM materials (collagen) have been widely applied in tissue engineering but hardly offer tissue-specific mechanical microenvironment and bioactive cues [15]. Interestingly, removing cellular components, not only are 3D structures and mechanical characteristics retained but also bioactive macromolecules (fibrous proteins and glycoproteins) and growth factors are remained in the dECM. Compared with single ECM component (Fibronectin, collagen I and laminin) used as the bioink to fabricate micropattern array chips, the dECM containing multiplex bioactive macromolecules can provide mimicking in-vivo tissue-specific matrix and microenvironment to regulate the cell viability, proliferation, differentiation, function, migration and organization [14], [15], [16].

## Ethics statements

All experimental protocols were approved by the Institutional Animal Care and Use Committee (IACUC) and Animal Experiment Center of Sichuan University (Approval No. 2021972A and 20220302064) and met the National Institutes of Health guide for the care and use of laboratory animals (NIH Publications No. 8023, revised 1978). All animals were cared for in accordance with the requirements of the Laboratory Animal Welfare Act and amendments thereof. Male Bama miniature pigs weighing 30–40 kg were used to carry out experiments. There was no observed difference of sex on the results of preparation of the dECM.

## CRediT authorship contribution statement

**Xinglong Zhu:** Methodology, Visualization, Investigation, Writing – original draft. **Yi Li:** Methodology, Visualization, Investigation, Writing – original draft. **Hulin Long:** Validation. **Zuoyu Liang:** Validation. **Yuting He:** Validation. **Yanyan Zhou:** Validation. **Shun Li:** Validation. **Ji Bao:** Conceptualization, Supervision, Writing – review & editing.

## Declaration of Competing Interest

The authors declare that they have no known competing financial interests or personal relationships that could have appeared to influence the work reported in this paper.

## Data Availability

No data was used for the research described in the article.
